# Secondary Metabolites from Two Species of *Tolpis* and Their Biological Activities

**DOI:** 10.3390/molecules171112895

**Published:** 2012-11-01

**Authors:** Jorge Triana, Mariana López, Francisco Javier Pérez, Milagros Rico, Aroa López, Francisco Estévez, María Teresa Marrero, Ignacio Brouard, Francisco León

**Affiliations:** 1 Departamento de Química, Unidad Asociada al CSIC, Universidad de Las Palmas de Gran Canaria, Campus de Tafira, 35017, Las Palmas de Gran Canaria, Spain; Email: mlopez@dqui.ulpgc.es (M.L.); fperez@dqui.ulpgc.es (F.J.P.); mrico@dqui.ulpgc.es (M.R.); aroa2412@gmail.com (A.L.); 2 Departamento de Bioquímica, Unidad Asociada al CSIC, Universidad de Las Palmas de Gran Canaria, Plaza Dr. Pasteur s/n, 35016, Las Palmas de Gran Canaria, Spain; Email: festevez@dbbf.ulpgc.es (F.E.); mariatemarrero@gmail.com (M.T.M.); 3 Fundación Canaria Instituto Canario de Investigación del Cáncer, Torre Agustín Arévalo-7ª Planta (PCTT-ULL) Avda de la Trinidad s/n 38204 San Cristóbal de La Laguna-S/C de Tenerife, Spain; 4 Instituto de Productos Naturales y Agrobiología, Consejo Superior de Investigaciones Científicas (CSIC), Avda. Astrofísico F. Sánchez, 3, 38206 La Laguna, Spain; Email: ibrouard@ipna.csic.es

**Keywords:** Asteraceae, *Tolpis proustii*, *Tolpis lagopoda*, ursane-triterpenes, taraxastane-triterpenes

## Abstract

Phytochemical research of two *Tolpis* species, *T. proustii* and *T. lagopoda*, led to the isolation of three new compounds: 30-chloro-3β-acetoxy-22α-hydroxyl-20(21)-taraxastene (**1**), 3β,22α-diacetoxy-30-ethoxy-20(21)-taraxastene (**2**) and 3β,28-dihydroxy-11α-hydroperoxy-12-ursene (**3**). The structures of the new compounds were elucidated by means of extensive IR, NMR, and MS data and by comparison of data reported in the literature. The *in vitro* antioxidant activities of the extracts were assessed by the DPPH and ABTS scavenging methods. The cytotoxicity of several known compounds and its derivatives was also assessed against human myeloid leukemia K-562 and K-562/ADR cell lines.

## 1. Introduction

The *Tolpis* genus (Asteraceae: Cichorioideae, Cichoriinae) consists of some 20 species distributed throughout Europe, North Africa, Canary Islands, Cape Verde and Asia, this genus being represented in the Canary Islands by around ten species [1].

Although a wide-ranging study of different species of *Tolpis* from the Canary Islands and from other Macaronesian archipelagos has been carried out from the botanical point of view [2,3], only one phytochemical report has previously appeared on the isolation and structural elucidation of aromatic compounds, triterpenes, and sterols from *T. webbi and T. spp* [4].

As a part of our continuing search for novel, plant-derived biological agents and our systematic investigation of the composition of Canarian endemic plants, the present work describes the isolation and structural elucidation of the constituents of the ethanolic extracts of the aerial parts of *T. proustii* Pitard in Pitard and Proust and *T. lagopoda* C.Sm. ex Buch. The constituents of these extracts were purified by CC, MPLC and preparative TLC. The structures of the known compounds were confirmed by comparison of their spectroscopic data with those reported in the literature.

**Figure 1:**
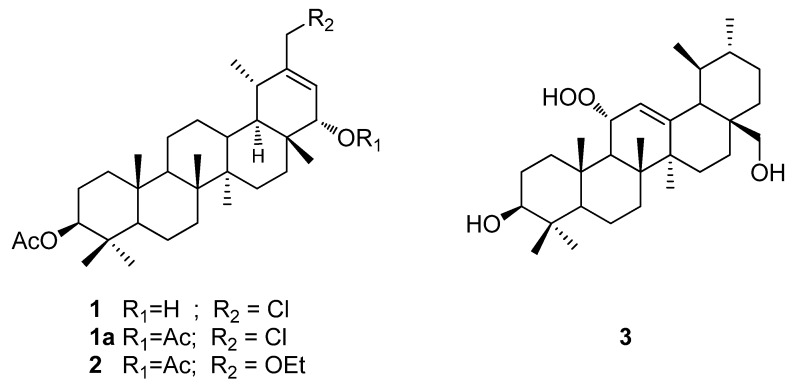
Compounds **1**–**3** isolated of the *T. proustii* and *T. lagopoda*.

From *T. proustii* seventeen compounds were isolated including two taraxan-triterpenoid 30-chloro-3β-acetoxy-22α-hydroxyl-20(21)-taraxastene **1**, and 3β,22α-diacetoxy-30-ethoxy-20(21)-taraxastene **2** a new ursan-triterpenoid 3β,28-dihydroxy-11α-hydroperoxy-12-ursene **3**. From *T. lagopoda*, eight compounds were isolated including a 3β,22α-diacetoxy-30-ethoxy-20(21)-taraxastene **2** (Figure 1).

The high level of solar radiation and the high temperature prevailing in the region of the Canary Islands (opposite the northwest coast of Africa) force plants to develop defence mechanisms against ultraviolet radiation and excessive production of free radicals through the accumulation of antioxidant metabolites [5]. This prompted us to evaluate the antioxidant activity of the crude extract of *T. proustii* and *T. lagopoda*, and that of some of the isolated compounds. Furthermore, one of the most important mechanisms by which tumor cells resist to cytotoxic effects of a variety of chemotherapeutic drugs is overexpression of the human multidrug resistance (MDR1) gene and its product, P-glycoprotein [6]. Here we have evaluated the effects of natural compounds and derivatives on the growth of the human leukemia K-562 and the P-glycoprotein-overexpressing K-562/ADR cell lines.

## 2. Results and Discussion

### 2.1. Structure Elucidation of Compounds ***1**–**3***

The positive EIMS spectrum of compound **1** showed a molecular ion at *m/z* 518/520 (3:1) in agreement with the presence of a chlorine atom in the structure, and with the formula C_32_H_51_O_3_Cl for this compound. In the same spectrum, the ions at *m/z* 500/502 [M-H_2_O]^+^, 458/460 [M-CH_3_COOH]^+^ suggested that this compound contained a hydroxyl and an acetyl group, respectively. Confirmed by the IR spectrum with absorptions of a hydroxyl 3446 cm^−1^ and a carbonyl 1730 cm^−1^ group. Its HREIMS experiment indicated the molecular formula C_32_H_51_O_3_^37^Cl (calcd. for [M]^+^ 520.3497; found 520.3497) and C_32_H_51_O_3_^35^Cl (calcd. for [M]^+^ 518.3527; found 518.3517). All spectral data suggested that **1** was a pentacyclic triterpene with a trisubstituted double bond in the E ring [7] with a 20(21)-taraxastane structure. 

**Figure 2:**
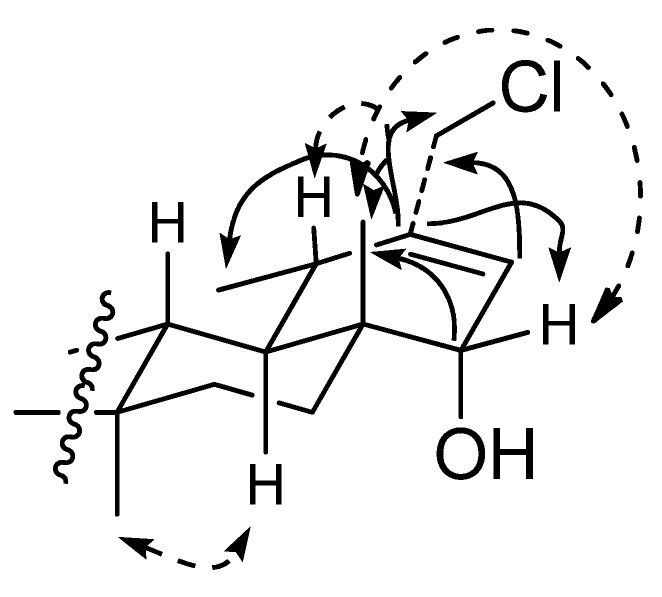
Selected correlations of **1**. Double-ended arrows indicate NOESY, and single arrows indicate HMBC (C to H) correlations.

The ^1^H and ^13^C-NMR (Table 1) spectra of **1** showed the presence of the oxygenated methine proton at δ_H_ 3.35 (1H, d, *J* = 6.6 Hz) and an unusual chloro atom at C-30 at δ_C_ 47.6 (Table 1). The structure elucidation and NMR assignments were therefore based primarily on the results of COSY, HSQC, HMBC, and NOESY experiments (Figure 2).

**Table 1:** ^1^H- and ^13^C-NMR data for compounds **1**, **1a** and **2***^a^*.

	1		1a		2	
Position	δ_H_	δ_C_	δ_H_	δ_C_	δ_H_	δ_C_
1	1.65 *	38.5	1.65 *	38.7	1.60 *	38.5
2	1.50 *	23.7	1.54 *	21.7	1.55 *	23.7
3	4.41 dd (6.1,10.8)	81.0	4.43 dd (6.4,10.0)	81.1	4.43 dd (5.1 10.4)	81.0
4	-	38.3	-	38.0	-	37.8
5	0.75 *	55.4	0.74 *	55.6	0.75 *	55.4
6	1.45 *	18.2	1.44 *	18.0	1.30 *	18.2
	1.35 *		1.34 *			
7	1.35 *	34.2	1.34 *	34.4	1.35 *	34.2
8	-	41.2	-	41.3	-	41.1
9	1.25 *	50.3	1.26 *	50.6	1.25 *	50.4
10	-	37.1	-	37.3	-	37.1
11	1.20 *	21.6	1.21 *	21.4	1.20 *	21.6
12	1.60 *	27.6	1.60 *	27.7	1.58 *	27.5
	1.20 *					
13	0.95 *	38.6	0.95 *		1.66 *	38.6
14	-	42.3	-	42.4	-	42.3
15	1.72 *	26.7	1.71 *	29.9	1.50 *	26.6
	1.05ddd (2.5,4.0,13.05)		1.02 *			
16	0.95 *	29.7	0.95 *	29.9	1.60 *	29.9
	1.85 dt (9.0,13.0)					
17	-	37.8	-	37.4	-	37.2
18	1.45 *	40.5	1.44 *	41.5	1.45 *	41.5
19	2.00 t (7.0)	31.6	2.03 tbr (7.0)	31.7	1.79 q (6.6)	32.3
20	-	144.9	-	146.5	-	147.7
21	5.89 d (6.5)	126.3	5.87 d (6.4)	123.1	5.75 d (6.3)	119.2
22	3.35 dbr (6.6)	73.3	4.51 d (6.4)	75.3	4.55 d (6.3)	75.3
23	0.78 s	28.0	0.79 s	28.1	0.81 s	28.0
24	0.77 s	16.6	0.78 s	16.7	0.80 s	16.5
25	0.82 s	16.4	0.82 s	16.7	0.81 s	16.4
26	0.99 s	16.1	0.98 s	16.2	0.96 s	16.1
27	0.93 s	14.7	0.91 s	14.7	0.92 s	14.6
28	0.63 s	17.8	0.70 s	17.9	0.70 s	18.2
29	0.99 d (6.5)	22.2	0.97 d (7.6)	22.1	0.96 d (6.6)	22.6
30	4.15 d (11.2)	47.6	4.14 d (11.2)	47.5	3.96 d (12.6)	72.3
	3.89 d (11.2)		3.89 d (11.6)		3.72 d (12.6)	
OH	3.14 m					
OAc	1.97 s	21.4	1.97 s	21.3	1.97 s	21.3
		171.4	1.98 s	21.3	1.98 s	21.3
				171.1		171.1
				171.1		171.1
OEt					1.13 t (6.9)	15.2
					3.35 m	65.9

*^a^* δ in ppm and *J* (in Hz) are in parentheses. Recorded in CDCl_3_ at 400 MHz and 125 MHz for ^1^H and ^13^C-NMR, respectively. * overlapped.

The most important HMBC and NOESY correlations are shown in Figure 1. Treatment of **1** with Ac_2_O-pyridine gave a diacetyl derivative **1a** for which its HREIMS experiment indicated the molecular formula C_34_H_53_O_4_^37^Cl (calcd. for [M]^+^ 562.3603; found 562.3624) and C_34_H_54_O_4_^35^Cl (calcd. for [M]^+^ 560.3654; found 560.3644). The data implied the presence of a double bond between C-20 and C-21, the chloro atom at C-30, and the acetyl group at C-3 and hydroxyl group at C-22. H-22 was assigned in β-orientation on the basis of the coupling constant with the vinylic proton at C-21 and cross-peak in the NOESY experiment with the CH_3_-28. From the above findings, the structure of 30-chloro-3β-acetoxy-22α-hydroxyl-20(21)-taraxastene was assigned to **1**, and it was named chlorotolpidiol. To the best of our knowledge, this is the first example of a pentacyclic triterpene of the taraxastane-ursane series with a chloro functionality.

Compound **2** was obtained as a colourless amorphous solid and its molecular formula was determined by a HRESIMS experiment as C_36_H_58_O_5_ (calcd. for [M+Na]^+^ 593.4182; found 593.4176). The IR spectrum revealed the absorption bands for a carbonyl group (1732 cm^−1^) and double bond (2872 cm^−1^). The ^1^H-NMR spectrum (Table 1) exhibited six singlet methyl groups at δ_H_ 0.70, 0.80, 0.81 (6H), 0.92 and 0.96, a secondary methyl group at δ_H_ 0.96 (3H, d, *J* = 6.6 Hz) attributed to C-29, a vinyl proton at δ_H_ 5.75 (1H, d, *J* = 6.3 Hz), two acetyl groups at δ_H_ 1.97 s and 1.98 s, two oxymethine signals at δ_H_ 4.43 (1H, dd, *J* = 5.1, 10.4 Hz) and 4.55 (1H, d, *J* = 6.3 Hz), an oxymethylene signal at δ_H_ 3.96 (1H, d, *J* = 12.6 Hz) and 3.72 (1H, d, *J* = 12.6 Hz) and an ethoxy group at δ_H_ 3.35 (2H, m) and 1.13 (3H, t, *J* = 6.9 Hz). The extra acetyl signal at C-22 was observed, since the oxygenated methine proton of **1** at δ_H_ 3.35 (1H, d, *J* = 6.6 Hz) was displaced to δ_H_ 4.55 (1H, d, *J* = 6.3 Hz) in **2**. Moreover, the halogenated group was replaced by a ethoxyl group at C-30 since the carbon in **2** was displaced to low field at δ_C_ 72.3 (Table 1). The structure of **2** was determined by a combination of COSY, DEPT, HSQC, HMBC, and NOESY experiments. Based on the above data, the new compound tolpidiol A **2** was identified as 3β,22α-diacetoxy-30-ethoxy-20(21)-taraxastene.

Compound **3 **was purified as its diacetate **3a** by treatment with acetic anhydride (Ac_2_O) in pyridine, **3a** was isolated as a colourless amorphous solid and its HRESIMS experiment indicated the molecular formula C_34_H_54_O_6_ (calcd. for [M+Na]^+^ 581.3818; found 581.3801). The IR spectrum revealed the absorption bands for carbonyl 1728 cm^−1^, and hydroxyl 3391 cm^−1^ groups, the presence of these groups was confirmed by the ^1^H and ^13^C-NMR spectra (Table 2). The ^1^H-NMR spectrum of **3a** showed signals for five tertiary methyl groups at δ_H_ 0.81(6H, br, s), 1.00, 1.02 and 1.11, and two secondary methyl groups at δ_H_ 0.86 (3H, d, *J* = 6.4 Hz) and δ_H_ 0.88 (3H, d, *J* = 7.3 Hz) suggesting a pentacyclic triterpene with an ursane skeleton. An olefinic proton at δ_H_ 5.30 (1H, d, *J =* 3.1 Hz) was assigned to H-12, two oxygenated methines at δ_H_ 4.45 (1H, dd, *J* = 3.0, 9.6 Hz) and 4.46 (1H, dd, *J* = 5.0, 9.5 Hz) corresponding to H-3 and H-11 respectively, the latter showing vicinal correlation in the COSY experiment with the olefinic proton H-12, while the proton H-9 δ_H_ 1.81 (1H, d, *J* = 9.5 Hz) indicated the presence of a hydroperoxide at C-11. The presence of an oxygenated methylene was confirmed by the signals at δ_H_ 3.56 (1H, d, *J* = 11.0 Hz) and 3.93 (1H, d, *J* = 11.0 Hz). The ^13^C-NMR (Table 2) and DEPT data indicated the presence of two ester carbonyl groups, nine methyl carbons, nine aliphatic methylenes, two olefinic carbons, and seven methine carbons. Thus, the position of acetyl groups in compound **3a** was assigned by a HMBC correlation between the signal at δ_C_ 171.0 and that at δ_H_ 4.45; and the signals at δ_C_ 171.3 and δ_H_ 3.93. The coupling constant between H-9 and H-11 (*J* = 9.5 Hz) established the α-orientation of the hydroperoxide at C-11. The EI-MS data of **3a** showed direct loss of H_2_O *m/z* 540 and H_2_O_2_ *m/z* 524, confirming the presence of the hydroperoxide.

**Figure 3:**
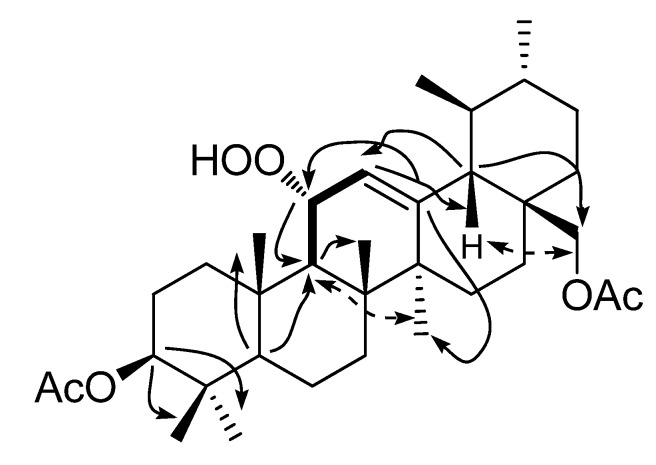
Selected correlations of **3a**. Bond bolded indicate COSY, Double-ended arrows indicate NOESY, and single arrows indicate HMBC (C to H) correlations.

The structure elucidation and NMR assignments were based primarily on the results of HSQC, HMBC, and COSY experiments which allowed the complete assignment of all H- and C-atoms, and the NOESY (Figure 3) data provided the configuration of compound **3a**. Therefore, the structure of **3** was established as 3β,28-dihydroxy-11α-hydroperoxy-12-ursene. To the best of our knowledge, compound **3** is a novel triterpenoid, which we named tolpidiol B.

**Table 2:** ^1^H and ^13^C-NMR data for compound **3a***^a^*.

Position	δH	δC
1	0.85 m	39.4
	2.08 dt (3.5, 7.0)	
2	1.58 *	23.7
3	4.45 dd (3.0,9.6)	80.6
4	-	38.0
5	0.88 m	55.3
6	1.45 *	18.1
	1.31 *	
7	1.45 m	33.3
	1.25 m	
8	-	43.2
9	1.81 d (9.5)	48.8
10	-	37.8
11	4.46 dd (5.0, 9.5)	81.6
12	5.30 d (3.1)	125.8
13	-	144.5
14	-	42.0
15	1.60 *	26.2
	0.90 m	
16	1.16 *	23.3
	1.92 td (3.5,9.0)	
17	-	36.9
18	1.45 m	53.7
19	1.35 m	39.0
20	1.28 m	39.3
21	1.40 m	30.4
22	1.31 m1.52 dt (3.0, 6.5)	35.5
23	0.81 s	28.2
24	0.81 s	16.7
25	1.02 s	16.8
26	1.00 s	18.0
27	1.11 s	22.2
28	3.56 d (11.0)	71.0
	3.93 d (11.0)	
29	0.86 d (6.4)	17.4
30	0.88 d (7.3)	21.3
OAc		21.0
	1.97 s	21.3
	1.97 s	171.0
		171.3

*^a^* δ in ppm and *J* (in Hz) are in parentheses. Recorded in CDCl_3_ at 400 MHz and 125 MHz for ^1^H- and ^13^C-NMR, respectively. * overlapped.

**Figure 4:**
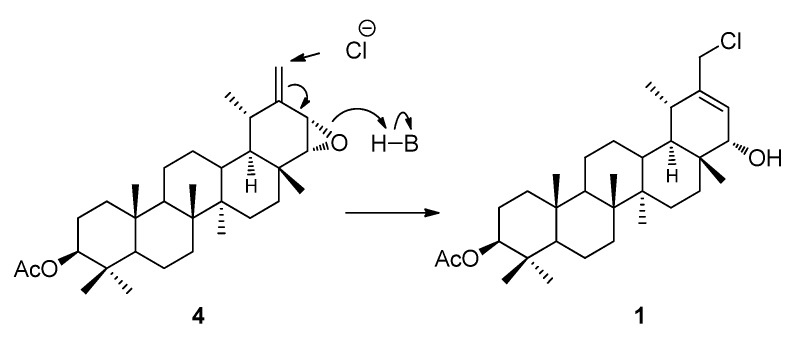
Tentative forming process of compound **1**.

Compound **1** could derive from the known triterpene acetyl-ptiloepoxide **4** [8,9] which was identified by us from *T. proustii* as an inseparable mixture. Triterpenes containing an epoxide at the Δ^21-22 ^position are known and have been isolated before from a *Tolpis* species [4]. Based on this, we envisioned the formation of compound **1** by chlorination of the double bond, followed by isomerization and β opening of the epoxide, and protonation, maintaining the α-orientation at the C-22 observed in the precursor compound acetyl-ptiloepoxide (Figure 4).

Although the number is relatively small, several halogenated triterpenes and other higher terpenes have been described mainly from marine sources [10]. However, the presence of chlorinated triterpenoids in terrestrial plants is very rare and just few cases have been reported [11,12,13,14]. Initially, compound **1** seemed to be an artifact of the isolation process, but Chen *et al.* [14] proved that to obtain chlorinated compounds, a chlorine source such as CHCl_3_ with HCl is necessary. During the isolation process, no chlorinated solvents were used (see Experimental). In the chromatographic separation, dichloromethane was used in the preparative TLC, which was not enough to interact with the possible precursor (ptiloepoxide) of **1**. Compound **2** was isolated as a presumed artifact; this compound was probably obtained from **1**, by a nucleophilic substitution reaction due to the use of hot EtOH during the extraction process. 

Additionally, from *T. proustii* 15 known compounds were isolated, including aromatic compounds: scopoletin [15] aesculetin [15] and apigenin [16]; the diterpene phytene-1,2-diol [17] and the triterpenoids stigmasterol [18], ergosterol peroxide [19], ursolic acid [20], lupan-20(29)-ene-3β,30-diol [21], 21α-hydroxytaraxasterol [22], 11-oxo-β-amyrin [23], 3β-acetoxy-urs-12-ene-1β,11α-diol [24], 21α,22α-epoxy-20α-hydroxy-20(30)-dihydrotaraxasterol [4], 3β-acetoxy-21α,22α-epoxytaraxastan-20α-ol [25], 22-oxo-20-taraxasten-3β-ol [9], β-amyrin [26]. From *T. lagopoda* seven known compounds were isolated, including aromatic compounds: 2,4′-dihydroxy-4-methoxybenzophenone [4] and triterpenoids: stigmasterol [18], ergosterol peroxide [19], a mixture of 7-oxo-β-sitosterol and 7-oxo-stigmasterol [26,27], ursolic acid [20], and α-amyrin [28]. Their structures were confirmed by comparison of their spectral data with those reported in the literature. 

### 2.2. Antioxidant Activities

Natural antioxidants that are present in plants are responsible for inhibiting or preventing the deleterious consequences of oxidative stress. In Table 3 the relative antioxidant efficiency of both *Tolpis* extracts against the DPPH radical is shown. Antioxidants suppress the absorbance at 515 nm on a time scale dependent on the antioxidant activity of extracts. The RSA of the crude extract of *T. proustii* (59.6%) was higher than that of *T. lagopoda* (41.4%). FRAP assay was used to study the ability of the antioxidants in the extracts to reduce ferric iron to the ferrous form. The same behaviour as for the DPPH assay was observed, *T. lagopoda* being less active than *T. proustii* (4.1 and 18.1 µmol of Fe(III) reduced to Fe(II) per gram of dry plant respectively) (Table 3). On the other hand, the free radical scavenging and ferric reducing power assays revealed that aesculetin (isolated from *T. proustii*) showed the highest antioxidant activities as compared with those of α-tocopherol and BHA (Table 4). Aesculetin gave a RSA value of 100% with a t_1/2_ (time required for 50% scavenging of DPPH radical in the specified concentration of antioxidant) of 22.5 seconds, while BHA and α-tocopherol showed RSA of 21.9 and 17.7% respectively after 20 min. Aesculetin (at concentration 0.1 mg mL^−1^) showed also higher antioxidant activities than both extracts (at concentration 10 mg mL^−1^), because the extracts are complex mixtures that include active components at lower levels. Moreover, the crude extracts tend to have more interfering substances that may interact with the antioxidants, decreasing their effectiveness. The antioxidant activities found in this study indicated that aesculetin, as well as both extracts, are ideal for use in the health food industry. Because of the high content of aesculetin found in the *T. proustii* extract (566.8 mg), this extract may be considered to be a natural source of aesculetin with diverse potential therapeutic uses. 

**Table 3:** Antioxidant activity of crude extracts derived from *T. proustii* and *T. lagopoda*.

Assays	*T. proustii*	*T. lagopoda*
RSA ^a^	59.6 ± 0.4	41.4 ± 0.1
FRAP ^b^	18.1 ± 0.4	4.1 ± 0.2
FRAP ^c^	93 ± 2	41 ± 1

^a^ % inhibition ± standard deviation of three measurements. ^b^ µmol of Fe(III) reduced to Fe(II) per gram of dry plant ± standard deviation of three measurements. ^c^ µmol of Fe(III) reduced to Fe(II) per gram of ethanolic residue ± standard deviation of three measurements.

**Table 4:** Antioxidant activity of aesculetin, α-tocopherol and butylated hydroxyanisol (BHA).

Assays	Aesculetin	α-Tocopherol	BHA
	0.1 mg mL^−1^	0.1 mg mL^−1^	0.1 mg mL^−1^
DPPH ^a^	100 ± 0	17.7 ± 0.1	21.9 ± 0.6
FRAP ^b^	9.4 ± 0.7	0.97 ± 0.03	3.13 ± 0.05

^a^ % inhibition ± standard deviation of three measurements. ^b^ µmol of Fe(III) reduced to Fe(II) per mg of compound ± standard deviation of three measurements.

### 2.3. Cytotoxic Activity

K-562 and K-562/ADR cells which are sensitive or resistant to doxorubicin, respectively, were incubated with the compounds shown in Table 5 to evaluate their potential cytotoxicity. After 72 h, cell survival was determined by the MTT assay and the IC_50_ values are summarized in Table 5. Among the different compounds ursolic acid and 22-oxo-20-taraxasten-3β-ol exhibit the strongest effects in mitochondrial reduction of tetrazolium salts to formazan, while the ursolic derivatives and the 1,2-diacetoxyphytene exhibit the weakest effects. Furthermore, K-562 and K-562/ADR cells exhibit comparable sensitivity to compounds ursolic acid and 22-oxo-20-taraxasten-3β-ol (Table 5). These results suggest that the overexpression of the drug efflux protein, P-glycoprotein does not confer resistance against these compounds.

**Table 5:** Effects of some compounds and derivatives isolated from *T. proustii* and *T. lagopoda* on the growth of the human leukemia cell lines.

Compound	IC_50_ (μM)	
	K562	K562/ADR
Ursolic acid	40.6 ± 3.6	49.2 ± 3.1
Ursolic acid methyl ester	59.3 ± 15.5	64.0 ± 14.5
Acetyl ursolic acid	99.5 ± 20.5	>100
Acetyl ursolic acid methyl ester	>100	>100
Aesculetin	63.2 ± 3.2	77.0 ± 5.1
Aesculetin acetyl	68.6 ± 17.1	70.3 ± 19.2
Aesculetin diacetyl	62.3 ± 6.6	59.5 ± 4.5
11-Oxo-β-amyrin	>100	>100
22-Oxo-20-taraxasten-3β-ol	30.0 ± 10.0	43.0 ± 7.0
1,2-Diacetoxyphytene	>100	>100

Cells were cultured for 72 h and the IC_50_ values were calculated as described in the Experimental section. The data shown represent the means ± SEM of 3–5 independent experiments with three determinations in each.

## 3. Experimental

### 3.1. General Experimental Procedures

Optical rotations: Perkin-Elmer *model 343* polarimeter. IR Spectra: Bruker model *IFS-55* spectrophotometer. ^1^H and ^13^C-NMR spectra: Bruker model AMX-500 and AMX-400 spectrometers with standard pulse sequences, operating at 500 and 400 MHz for ^1^H-, and 125 MHz for ^13^C-NMR, CDCl_3_ was used as solvent and TMS as internal standard. EI–MS: *Micromass* model *Autospec* (70 eV) spectrometer. The constituents of the ethanolic extracts were separated by gravity column chromatography, medium pressure liquid chromatography (MPLC) and preparative TLC. Column chromatography (CC): silica gel SiO_2_; (70–230 mesh, Merck), column fractions were monitored by TLC (silica gel *60 F_254_*), Medium pressure column chromatography (MPLC): silica gel *Merck* (40–63 μm). Prep. TLC: silica gel *60 PF_254 + 366_* plates (20 × 20 cm, 1-mm thickness).

### 3.2. Plant Material

The plant material was identified by Dr. Rosa Febles. *T. proustii* was collected at Roque Agando (La Gomera, Canary Islands) in May 2009. A voucher specimen has been deposited at the Herbarium of the Viera y Clavijo Botanical Garden in Las Palmas de Gran Canaria (LPA 24194). *T. lagopoda* was collected at Tenteniguada (Gran Canaria, Canary Islands) in May 2008. A voucher specimen has been deposited at the Herbarium of the Viera y Clavijo Botanical Garden in Las Palmas de Gran Canaria (LPA 23596).

### 3.3. Extraction and Isolation

The aerial parts of *T. proustii* (3.0 Kg) were extracted with ethanol (4 L) in a Soxhlet until exhaustion. Solvent removal afforded a viscous residue (541.5 g) which was fractionated by means of CC (SiO_2_; hexane/AcOEt step gradients). The fractions eluted with hexane-AcOEt (7:3) were submitted to a new MPLC chromatography with hexane-AcOEt (9:1), to give stigmasterol (30.6 mg); 3β-acetoxy-21α,22α-epoxy-20α-hydroxy-20(30)-dihydrotaraxasterol (6.3 mg), obtained by preparative TLC (benzene-ethyl acetate 8:2); ergosterol peroxide (obtained as its acetylated derivative upon acetylation of one of the obtained fractions (2.3 mg); 3β,30-dihydroxylup-20(29)-ene (31.1 mg); phytene-1,2-diol (7.6 mg); **3a** (2.3 mg), isolated by successive preparative TLCs (benzene-AcOEt, 9.9:0.1, 2 elutions; dichloromethane-hexane 1:1, 3 elutions) and **1** (3.7 mg), obtained by preparative TLC (dichloromethane, 3 elutions). Subsequent chromatography by MPLC with hexane-AcOEt (8.5:1.5) gave ursolic acid (193.8 mg); 21α-hydroxytaraxasterol (8,9 mg), obtained by preparative TLC (benzene-AcOEt, 8.5:1.5, 4 elutions); 11-oxo-β-amyryn (15.4 mg); 22-oxo-taraxasten-3β-ol (2.7 mg) isolated by preparative TLC (dichloromethane-AcOEt 9:1, 4 elutions); β-amyryn (5.2 mg); and **2 **(2.1 mg), obtained upon crystallization in hexane-AcOEt and subsequent purification by preparative TLC (dichloromethane-AcOEt 8:2).

The fractions eluted with hexane-AcOEt (3:2) were partially rechromatographed by MPLC in hexane-AcOEt (7:3) giving, after preparative TLC (benzene-AcOEt, 8:2, 2 elutions), apigenin (41.2 mg) and scopoletin (6.7 mg). The remainder of these fractions was submitted to subsequent rechromatography by MPLC in hexane-AcOEt (8.5:1.5) to give 3β-acetoxy-1β,11α-dihydroxy-urs-12-ene (9.7 mg), isolated by preparative TLC (benzene-AcOEt, 8:2, 4 elutions), and 21α,22α-epoxy-20α-hydroxy-20(30)-dihydrotaraxasterol (3.6 mg), purified by preparative TLC (hexane-AcOEt, 8:2, 2 elutions). Finally, the fractions eluted with hexane-AcOEt (1:1) afforded aesculetin (566.8 mg), upon crystallization in hexane-AcOEt.

The aerial parts of *T. lagopoda* (2.7 Kg) were extracted with ethanol (4 L) in a Soxhlet until exhaustion. Removal of the solvent afforded a viscous residue (279.8 g), which was fractionated by CC (SiO_2_; hexane/AcOEt step gradients). Fractions eluted with hexane-ethyl acetate (4:1) were subsequently chromatographed by MPLC with hexane-ethyl acetate (7.5:2.5) to give five fractions (numbered from fraction 1 to 5). From fraction 1 stigmasterol (14.3 mg) was purified by crystallization in hexane-AcOEt, while fraction 3 afforded 2,4′-dihydroxy-4-methoxybenzophenone**,** obtained by crystallization with hexane-AcOEt (15.1 mg) and from its mother liquors α-amyrin (2.9 mg) was obtained by preparative TLC with (benzene-AcOEt, 9:1, 5 elutions), as its acetylated derivative, after acetylation*.* From fraction 4 ursolic acid (6.1 mg) was purified after a methylation process. Fraction 5 afforded ergosterol peroxide (11 mg) by preparative TLC (hexane-AcOEt, 9:1, 2 elutions), purified after acetylation as its acetyl derivative. Finally, fraction 2 afforded compound **2** (1.8 mg) by preparative TLC (benzene-AcOEt, 9:1). Fractions eluted with hexane-AcOEt (7:3) were subsequently chromatographed by MPLC with hexane-AcOEt (8:2), the most polar fractions affording the mixture of 7-oxo-β-sitosterol and 7-oxo-stigmasterol (18.1 mg) were purified by preparative TLC (hexane-AcOEt, 7:3, 5 elutions). 

30-Chloro-3β-acetoxy-22α-hydroxyl-20(21)-taraxastene (**1**). Amorphous white solid; [α]^25^_*D*_ = +6.0 (CHCl_3_, c 0.001); for ^1^H and ^13^C-NMR data, see Table 1. IR, ν max (KBr): 3446, 2924, 2853, 1732, 1439, 1244, 1172 cm^−1^. EIMS *m/z* 520 (5.7), 518 (15.5), 502 (13.5), 500 (35.2), 483 (7.3), 460 (4.3), 458 (10.5), 464 (13.6), 404 (5.1) 249 (21.0), 190 (36.5) 189 (100.0), 187(40.0) 161 (17.0), 133(29.0) 121 (36.0), 81(31.6). HREIMS *m/z* 520.3497 [M]^+^ (calcd for C_32_H_51_O_3_^37^Cl 520.3497), 518.3517 [M]^+^ (calcd for C_32_H_51_O_3_^35^Cl 518.3527), 502.3414) [M−H_2_O]^+^ (calcd for C_32_H_49_O_2_^37^Cl 502.3392), 500.3436 [M−H_2_O]^+^ (calcd for C_32_H_49_O_2_^35^Cl 500.3421. 

Acetylation of **1**. Compound **1** (2.0 mg) was dissolved in pyridine (1 mL) and acetic anhydride (2 mL), and the reaction was further stirred at room temperature for 12 h and after usual work-up. The product was dried under vacuum to furnish **1a** (1.8 mg). For ^1^H and ^13^C-NMR spectroscopic data, see Table 1.

30-Chloro-3β,22α-diacetoxy-20(21)-taraxastene (**1a**). Amorphous white solid; [α]^25^_*D*_ = +86.7 (CHCl_3_, c 4.5 × 10^−3^); for ^1^H and ^13^C-NMR data, see Table 1. IR, ν max (KBr): 2920, 2852, 1731, 1652, 1540, 1456, 1372, 1247, 1016, 982 cm^−1^. EIMS *m/z* 562 (0.7), 560 (1.7), 525 (34.7), 500 (27.0), 466 (10.4), 404 (6.4), 189 (100), 95 (52.8), 69 (56). HREIMS m/z 562.3624 [M]^+^ (calcd for C_34_H_53_O_4_^37^Cl 562.3603), 560.3644 [M]^+^ (calcd for C_34_H_53_O_4_^35^Cl 560.3632).

3β,22α-Diacetoxy-30-ethoxy-20(21)-taraxastene (**2**). Colourless amorphous solid; [α]^25^_*D*_ = +4.8 (CHCl_3_, c 0.015); for ^1^H and ^13^C-NMR data, see Table 1. IR, ν max (KBr): 2931, 2872, 1732, 1652, 1464, 1456, 1671, 1244, 1016 cm^−1^. EIMS *m/z* 510 (100) [M−OAc]^+^, 450 (40) [M−2OAc]^+^. HRESIMS positive ion *m/z* 593.4176 [M+Na]^+^ (Calcd. for C_36_H_58_O_5_Na, 593.4182).

3β,28-Diacetoxy-11α-hydroperoxy-12-ursene **3a**. Colourless amorphous solid; [α]^25^_*D*_ = +9.5 (CHCl_3_, c 0.014); for ^1^H and ^13^C-NMR data, see Table 2. IR, ν max (KBr): 3391, 2951, 2925, 2875, 1728, 1392, 1244, 1032, 983, 903 cm^−1^. HREIMS *m/z* 540.3829 [M−H_2_O]^+^ (Calcd. for C_34_H_52_O_5_, 540.3815); m/z 524.3873 [M−H_2_O_2_]^+^ (Calcd. for C_34_H_52_O_4_, 524.3866). HRESIMS positive ion *m/z* 581.3801 [M+Na]^+^ (Calcd. for C_34_H_54_O_6_Na, 581.3818).

### 3.4. Antioxidant Experiments

#### 3.4.1. Chemicals

Methanol HPLC grade (Panreac, Barcelona, Spain) and Milli-Q water (18MQ, Millipore, Billerica, MA, USA) were always used in this study. Formic acid and sodium acetate (Merck, Darmstadt, Germany) were analytical quality reagents. 1,1-Diphenyl-2-picrylhydrazyl (DPPH) and 2,4,6-tri (2-pyridyl)-1,3,5-triazine (TPTZ) were purchased from Sigma-Aldrich Chemie (Steinheim, Germany); rutin and gentisic and caffeic acids were supplied by Merck. Ferric chloride (FeCl_3_·6H_2_O), ferrous sulphate (FeSO_4_·7H_2_O) and glacial acetic acid were obtained from Panreac.

#### 3.4.2. Preparation of Extracts for Antioxidant Assays

The ethanolic extract residue was solved in methanol by stirring at room temperature. After centrifugation at 7,000 rpm for 10 min, the supernatant was collected and the antioxidant activity was measured. Aesculetin, α-tocopherol and BHA (5 mg) were dissolved in methanol (10 mL). The solution was diluted to 0.1 mg mL^−1 ^to be used for antioxidant assays.

#### 3.4.3. Free Radical Scavenging Activity on DPPH

The reducing ability of antioxidants towards DPPH radical was evaluated by measuring the loss of 1,1-diphenyl-2-picrylhydrazyl (DPPH) colour at 515 nm after reaction with test extracts [29]. The sample solution (30 μL) was rapidly mixed with 1 mL of a 0.1 mM DPPH solution. After 25 min incubation time in the dark at ambient temperature (23 °C), the decline in absorbance against a methanol blank was recorded. The inhibition percentage values were calculated by equation: Radical Scavenging Activity (RSA) = 100 (1–Abs in the presence of sample/Abs in the absence of sample).

#### 3.4.4. Ferric Reducing Antioxidant Power Assay (FRAP)

Reducing power was determined according to [30]. This method is based on the reduction of Fe^3+^ to Fe^2+^, which is recorded by measuring the formation of a blue colored Fe^2+^-tripyridyltriazine compound from the colourless oxidized Fe^3+^ form by the action of electron donating antioxidants. The FRAP reagent consists of 300 mM acetate buffer (3.1 g sodium acetate + 16 mL glacial acetic acid, made up to 1 litre with distilled water; pH = 3.6), 10 mM TPTZ in 40 mM HCl and 20 mM FeCl_3_.6H_2_O in the ratio of 10:1:1.

Extract (10 μL) was added to 1.0 mL freshly prepared and prewarmed (37 °C) FRAP reagent. The mixture was incubated at 37 °C for 10 min and the absorbance was measured against a reagent blank (1.0 mL FRAP reagent + 10 μL distilled water) at 593 nm. A standard curve of Fe^2+^ was constructed over the concentration range of 0.2 µmol L^−1^ to 1 µmol L^−1^. The results were determined by the regression equation of the curve (y = 0.00221x + 0.00999, r = 0.9969) and expressed as µmol ferric ions reduced to ferrous form per g of dry plant material.

### 3.5. Cytotoxic Experiments

#### 3.5.1. Cell Culture

Human leukemia K-562 cells (DSMZ No: ACC 10, German Collection of Microorganisms and Cell Cultures, Braunschweig, Germany) were cultured in RPMI 1640 containing 2 mM L-glutamine supplemented with 10% (v/v) heat-inactivated fetal bovine serum. The K-562/ADR cell line was kindly provided by Professor Lisa Oliver (INSERM, Nantes, France) and cultured as above.

#### 3.5.2. Assay for Growth Inhibition and Cell Viability

The cytotoxicity of compounds was assessed using a 3-(4,5-dimethylthiazol-2-yl]-2,5-diphenyl-2*H*-tetrazolium bromide (MTT) assay. Briefly, 1 × 10^4^ exponentially growing cells were seeded in 96-well microculture plates with various compounds concentrations (3–100 μM) in a volume of 200 μL. DMSO concentration was the same in all the treatments and did not exceed 0.1% (v/v). After 72 h, surviving cells were detected based on their ability to metabolize 3-(4,5-dimethylthiazol-2-yl)-2,5-diphenyl-2*H*-tetrazolium bromide into formazan crystals. Optical density was read with an ELISA reader at a wavelength of 570 nm and was used as a measure of cell viability. The MTT dye reduction assay measures mitochondrial respiratory function and can detect the onset of cell death earlier than dye-exclusion methods. Cell survival was calculated as the fraction of cells alive relative to control for each point: cell survival (%) = mean absorbance in treated cells/mean absorbance in control wells × 100. Concentrations inducing a 50% inhibition of cell growth (IC_50_) were determined graphically using the curve fitting algorithm of the computer software Prism 4.0 (GraphPad). Values are means ± SEM from three independent experiments, each performed in triplicate.

## 4. Conclusions

We have studied two endemic Canary plants of the *Tolpis* genus and two new compounds tolpidiol A (**2**) and B (**3**) and a novel chloro derivative chlorotolpidiol (**1**) were isolated from *T. proustii* and *T. lagopoda*. The isolation of halogenated triterpenoids from terrestrial sources is very rare, and few cases have been reported. To the best of our knowledge, chlorotolpidiol is the first example of a pentacyclic triterpene of the taraxastane-ursane series chlorinated at the position C-30. Herein we present a tentative explanation of the biogenesis of this class of compounds. The presence of chlorine atoms provides chemically addressable handles for further work in organic-medicinal chemistry. The antioxidant activities of the ethanolic extract were evaluated and it showed relative efficiency. We found weak cytotoxic activity against K562 and K-562/ADR cell lines of some of the isolated and derivative compounds.
